# 'Reluctant pioneer': A qualitative study of doctors' experiences as patients with long COVID

**DOI:** 10.1111/hex.13223

**Published:** 2021-03-22

**Authors:** Anna K. Taylor, Tom Kingstone, Tracy A. Briggs, Catherine A. O'Donnell, Helen Atherton, David N. Blane, Carolyn A. Chew‐Graham

**Affiliations:** ^1^ School of Medicine Leeds Institute of Health Sciences Faculty of Medicine and Health, University of Leeds Leeds UK; ^2^ School of Medicine Faculty of Medicine and Health Sciences Keele University Keele UK; ^3^ Research and Innovation Department St George's Hospital Midlands Partnership NHS Foundation Trust Stafford UK; ^4^ Division of Evolution and Genomic Sciences School of Biological Sciences Manchester University Manchester UK; ^5^ General Practice & Primary Care Institute of Health & Wellbeing University of Glasgow Glasgow UK; ^6^ Unit of Academic Primary Care Warwick Medical School University of Warwick Coventry UK

**Keywords:** chronic disease, COVID‐19, doctors as patients, general practice, help‐seeking, long COVID, professional identity

## Abstract

**Background:**

The coronavirus disease (COVID‐19) pandemic has had far‐reaching effects upon lives, healthcare systems and society. Some who had an apparently 'mild' COVID‐19 infection continue to suffer from persistent symptoms, including chest pain, breathlessness, fatigue, cognitive impairment, paraesthesia, muscle and joint pains. This has been labelled 'long COVID'. This paper reports the experiences of doctors with long COVID.

**Methods:**

A qualitative study; interviews with doctors experiencing persistent symptoms were conducted by telephone or video call. Interviews were transcribed and analysis conducted using an inductive and thematic approach.

**Results:**

Thirteen doctors participated. The following themes are reported: making sense of symptoms, feeling let down, using medical knowledge and connections, wanting to help and be helped, combining patient and professional identity. Experiencing long COVID can be transformative: many expressed hope that good would come of their experiences. Distress related to feelings of being ‘let down’ and the hard work of trying to access care. Participants highlighted that they felt better able to care for, and empathize with, patients with chronic conditions, particularly where symptoms are unexplained.

**Conclusions:**

The study adds to the literature on the experiences of doctors as patients, in particular where evidence is emerging and the patient has to take the lead in finding solutions to their problems and accessing their own care.

**Patient and Public contribution:**

The study was developed with experts by experience (including co‐authors HA and TAB) who contributed to the protocol and ethics application, and commented on analysis and implications. All participants were given the opportunity to comment on findings.

## 1 BACKGROUND

The novel coronavirus disease (COVID‐19) pandemic declared by the World Health Organization in March 2020 has had far‐reaching effects upon people's lives, healthcare systems and wider society. Acute respiratory syndrome coronavirus 2 (SARS‐CoV‐2) causes dramatic variation in clinical outcome, from asymptomatic infection through to multi‐organ failure and death.^1^ In the United Kingdom (UK), over 190 000 people have been hospitalized with COVID‐19 as of November 2020, with 17% requiring intensive care and 26% of these people dying.^2^, ^3^ In June 2020, NHS England published '*After‐care needs of inpatients recovering from COVID‐19'*, estimating that up to that date, more than 95 000 patients had been admitted to hospitals across England with COVID‐19 and it assumed 45% would need ongoing support.^4^ Indeed, some estimates suggest that up to 50% of people hospitalized will need formal rehabilitation services.^5^

Some people who had an apparently 'mild' COVID‐19 infection (whether confirmed or suspected) continue to suffer from persistent symptoms, including fatigue, cognitive impairment ('brain fog'), neuropathy and paraesthesia, chest pain and palpitations, muscle and joint aches and shortness of breath.^6^, ^7^, ^8^, ^9^, ^10^, ^11^, ^12^, ^13^ This has been termed ‘long COVID’ by people with the persisting symptoms.^12^, ^14^, ^15^ The NICE scoping document defines this as post‐COVID‐19 syndrome.^16^ Evidence for the extent of long COVID is growing: for example, a team from Italy, one of the earliest affected countries in Europe, reported that 87% of people discharged from a Rome hospital were still experiencing at least one symptom 60 days after the onset of COVID‐19 and 55% had three or more symptoms including fatigue (53%), difficulty in breathing (43%), joint pain (27%) and chest pain (22%), with 40% reporting that it had reduced their quality of life.^9^ Public Health England published guidance on 7th September 2020 stating that around 10% of ‘mild’ COVID‐19 cases who were not admitted to hospital have reported symptoms lasting more than four weeks and a number of hospitalized cases reported continuing symptoms for eight or more weeks following discharge.^17^ Similarly, in a UK study (preprint) of a young, low‐risk cohort of 201 participants affected by COVID‐19, 70% were found to have organ damage with impairment in one or more organs four months after symptom onset; of note, 31% of participants were healthcare workers.^18^

From early in the pandemic, doctors highlighted the vulnerability of healthcare workers in terms of both physical health and mental health.^19^ Indeed, in the first wave, a sixth of admitted COVID‐19 cases were healthcare workers and their households; for patient facing healthcare workers, there was a threefold increased risk of admission with COVID‐19 compared with the general population.^20^ What is notable about long COVID is that several doctors, including those who have become patients with long COVID, have reflected publicly on their experiences.^7^, ^21^

Previous literature on doctors who become patients describes the need to be seen as a person, and treated as a ‘patient’, but at the same time, doctors experience a loss of professional identity when they become ill.^22^ They have also frequently reflected on the standards of care they have experienced and how they wanted to change clinical practice as a result.^22^ Doctors may be less likely to seek help than the general public due to structural barriers such as difficulties accessing care during normal working hours, or inconsistent primary care provision due to frequent moves.^23^ However, there may also be psychological barriers to help‐seeking with the perception that doctors cannot become ill, and the patient is ‘the one with the disease’.^24^ Additionally, doctors have been described as having certain personality traits including perfectionism and denigration of vulnerability, which may also prevent, or at least reduce, help‐seeking as they develop a ‘medical identity’ in which their professional responsibilities permeate all aspects of their lives.^23^, ^25^, ^26^

Those doctors who do have time out of work for health reasons often describe isolation, frequently experiencing stigma from colleagues and family as well as expressing self‐stigmatizing views of themselves as ‘failures’.^27^, ^28^ The limited literature suggests that it is hard to be a doctor and a patient. This may be particularly problematic when the condition you are experiencing is novel and poorly understood. This qualitative study reports the experiences of doctors with long COVID. The analysis of interviews reported here is part of a wider study,^29^ the aim of which was to explore the experiences of people with persisting symptoms following suspected or confirmed COVID‐19 infection in March or April 2020, which became known as ‘long COVID’ during the course of their illness.

## 2 METHODS

A qualitative study was conducted, using semi‐structured interviews with people with long COVID recruited through social media, to generate data.^29^ This study was developed in collaboration with experts by experience (people with persisting symptoms, or long COVID). Authors HA and TAB both have long COVID and thus are experts by experience; HA is a health services researcher and is married to a clinician, and TAB is a clinical academic. One of the authors (CCG) discussed the planned study with members of a post‐COVID support group, facilitated by a clinical commissioning group.

A subset of interviews were conducted with doctors experiencing persistent symptoms following COVID‐19 or suspected COVID‐19 infection. Semi‐structured interviews were chosen to allow participants to talk without limitation about the areas they felt were most important, while ensuring that all topics felt relevant by the research team were covered and perspectives were explored in detail.^30^, ^31^ The protocol and documents for University ethics application were developed in collaboration with experts by experience.

### 2.1 Recruitment

Doctors with self‐reported experiences of long COVID were recruited using social media posts (Facebook and Twitter). They were invited to contact the research team by email to express interest, and potential participants were sent a participant information sheet and consent form. Those doctors who agreed to participate in an interview were invited to pass on details of the study to others they knew who were experiencing similar symptoms, including sharing to peer support groups on social media (‘snowball sampling’).^32^

### 2.2 Data collection

Interviews were conducted by telephone or using software such as Microsoft Teams or Zoom, according to the participant's preference, during July and August 2020. Consent was obtained (consent form completed and sent by email to members of the research team) in advance of the interviews, reconfirmed and audio‐recorded at the start and end of the interview. Interviews were conducted by TK (social scientist) and CCG (academic GP), both experienced in qualitative research methods, who both kept reflective notes which contributed to analysis.

The topic guide was developed by the researchers in collaboration with experts by experience. The topic guide was modified iteratively throughout data collection and analysis.

Details including when acute COVID‐19 infection was experienced, whether infection was confirmed by antigen [Ag] or antibody [Ab] testing, and demographic information (including age, ethnicity, occupation) were from participants, in order to contextualize the data and support description of the sample.

Following the interview, participants were asked if they wished to receive a summary of the findings and/or publications arising from the study. They were also offered a voucher to compensate them for their time and were sent a ‘thank you’ email. Comments sent back by participants (by email) who reviewed the summary of findings were incorporated into the ongoing analysis.

Digital audio‐recordings of the interviews were transcribed by members of the research team, or a professional transcribing company.

### 2.3 Data analysis

Data analysis, following an inductive and interpretive thematic approach^33^, ^34^ and applying principles of constant comparison,^35^ was conducted by the research team. This form of analysis allows researchers to understand collective experiences of participants.^33^ The research team represented a mix of professional backgrounds, allowing for robust analysis of the data from differing perspectives. AKT and CCG analysed all transcripts; each coauthor analysed a subset. The codes, themes, and any alterations to the topic guide were discussed collectively and agreed upon by the research team. Sample size was determined by inductive thematic saturation, which was judged to be the point at which no new codes or themes were developed from the analysis.^36^, ^37^

## 3 RESULTS

Thirteen doctors were interviewed. Eleven participants were female and eleven White British. None were working at the time of their interviews due to their persisting symptoms. Four participants were aged 30‐39, five were aged 40‐49, and three were over the age of 50 (see Table 1 for other participant demographics).

**TABLE 1:** Participant demographics

Participant pseudonym	Employment status at time of coronavirus infection	Long‐term condition?	Month developed coronavirus symptoms (2020)	Ag test?	Ab test?
Alice	Not working	Yes	March	Negative	Negative
Beverley	GP	No	March	No	No
Caryn	Psychiatrist	Yes	March	Negative	Inconclusive
Deborah	GP	No	April	Negative	No
Edward	GP	No	March	Negative	No
Farhad	GP	No	March	Positive	Negative
Georgina	GP	Yes	March	Negative	Negative
Harriet	Medical educator	Yes	March	Negative	No
Irena	GP	Yes	March	Positive	No
Jenna	GP	Yes	March	No	No
Kate	Anaesthetist	No	April	Negative	No
Lisa	Anaesthetist	Yes	March	Negative	No
Moira	GP	No	End of April	Negative	Negative

Interviews lasted between 57 and 120 minutes (mean 87 minutes). The following themes will be presented in this paper, with illustrative quotes identified by a pseudonym for each participant: making sense of symptoms, feeling let down, using medical knowledge and connections, wanting to help and wanting to be helped and becoming a more empathic doctor.

### 3.1 Making sense of symptoms

Participants described both concern and confusion over the cause and nature of their symptoms, and the challenge of attempting to fit the symptoms they were experiencing into their own existing medical knowledge.

*‘It's weird because one minute, you can walk up the stairs and you can feel fine and the next minute, you walk up and all your muscles are killing you and your heart is batting at 120. It's just bizarre.’* Deborah

*‘I went through this cycle of feeling a bit better… I thought I’d gradually try and increase my exercise tolerance in my garden… but I’d get to the point where I literally couldn’t walk another step, it was like my battery had run out. It wasn’t just fatigue, it was the weirdest thing…’* Georgina

Participants described the unusual and unpredictable nature of their symptoms:

*‘I also had this really horrible sensation in my throat, which people have called COVID Strangle because it feels like you're being strangled. It was on and off and sometimes, say, I'd have it for the morning and then by afternoon, it had completely gone but it was very unpleasant. I also had severe acid reflux… I didn't get that strangle feeling again but I did have raised lymph nodes in my neck which ached a lot… One thing that I've had the entire time is shortness of breath and that's still bad now. One of the key things is when I'm talking to my children, I run out of breath. If I'm talking or reading them books, I run out of breath, which is strange because if I go for, say, a 15 minute walk, I can walk without feeling breathless. That's very strange.’* Alice

They feared that their symptoms represented serious pathology, such as pulmonary embolism or myocarditis, that had not been considered or investigated and which would cause them significant long‐term problems.

*‘If you’re getting cardio and respiratory symptoms, pathology needs ruling out before rehab.’* Harriet

Most participants expressed concern over whether they would ever recover:

*‘It’s difficult because I keep getting new things, which is one of the frustrations of this. The brain stuff seems to be getting better, to the level that I can function. When the brain wasn’t working that made me very scared because I need my brain! Not to be blasé, but the chest pain and stuff I can still work because I can work remotely. If I don’t have my brain I can’t work, I can’t plan, I can’t string a sentence together… I did get a bit scared when I was ill for so long… at the moment in a way I’m not missing out on a lot of stuff because everything is virtual.’* Irena

Symptoms persisting after the initial illness were not expected and even contradicted by initial ideas about SARS‐CoV‐2 from knowledge available in early 2020. Because of this, participants reported seeking advice, support and information from online groups, including specific groups for doctors with persisting symptoms, in order to try to make sense of their own symptoms as well as seeking reassurance that their symptoms were shared by other people.

*‘It’s been rather mysterious conditions and it doesn’t feel like anything else, it’s bizarre, and so even just to find other people say ‘yeah, I get that too, oh right, it must be part of it’ is helpful in understanding what’s going on.’* Harriet

Some participants, however, expressed fears that reading about symptoms experienced by other people could trigger those same symptoms in themselves, and described an internal debate over whether the origin of their own symptoms was physical or psychological.

*‘And now I feel okay but I’m just waiting for the next time I feel crap again. And then you think you might be making it worse because you’re like ‘now I’m kind of focussing too much on my body’ because before I’d think, ‘oh are my legs aching again is it happening?’ And then you start focussing on it and you think you might somehow think yourself into it.’* Beverley

Participants expressed fear that certain symptoms would be perceived negatively by their GP, and described selectively disclosing symptoms to avoid symptoms being dismissed or attributed to a potentially stigmatizing condition.

*‘I'd only admitted fatigue to my GP at that point, but I was also having episodes of quite significant tachycardia, I’d often get a heart rate of 130, just randomly watching TV. And avoided telling my GP about that I think for several reasons. I was also getting really tachycardic on any exertion. And I didn’t want to admit it because I think I was worried about it being dismissed as anxiety.’* Jenna

Participants described feelings of guilt at being ill, reflecting on the burden on their family and friends, but also on work colleagues, as illustrated by Edward who was not yet back at work as a GP:

‘*So I do my bit there, but I feel aggrieved that I’m not pulling my weight with the workload that’s now there. You know, it’s a lot. So that’s, that makes me feel quite guilty really because, you know, I can go out. I can get out and go for a walk with caution. Do that sort of stuff and, you know, I can go and visit relatives. I can’t drive yet. I’m not driving yet*…’ Edward

### 3.2 Feeling let down

Some participants reported that they had been advised by NHS 111^1^ or a GP to stay at home, when they were suffering from acute coronavirus symptoms, often in the face of symptoms that in non‐COVID‐19 times would have meant they would have used their experience and knowledge as a doctor and called an ambulance:

*‘…so I spoke to somebody else who I didn’t know at the practice. And she, I was coughing a lot and really short of breath and it was quite difficult for me to have sensible conversation because every time I tried to talk, I was just a massive cough monster. And she just asked me, she asked me if I wanted to be seen at the hot hub* 2 *and I said no to that because… I felt too ill to even think about getting myself there…’* Jenna

Participants expressed disappointment with the perceived lack of interest and support from doctors who they consulted, when it came to acknowledging and investigating their ongoing symptoms. They described how their expertise as a doctor with symptoms had not been recognized or taken seriously:

*‘And my GP wasn’t really very interested in it. I think at my kind of insistence she discussed with a medical consultant at the hospital, and the consultant said, “Well, that’s normal for COVID. That’s what people are experiencing, so there’s no investigations needed”, which to me didn’t feel remotely reassuring.’* Caryn

This need to apply pressure on their own GPs to obtain onward referral was commonly described but, as with Caryn above, the specialist opinion was not always the outcome they hoped for. Other participants described a negative response to requests for referrals:

*‘I wanted outpatient referral, I wanted an opinion from a specialist who knew about this, I wanted my tachycardia evaluating, I don’t know if it’s AF or what… I wondered if I could have some basic bloods. She said “there’s nothing we can do, this is all post‐viral and you need to just wait and rest”. I felt dismissed, I felt unheard, I felt angry, quite tearful at the time… There was no kindness in her voice. It was almost tick‐box, another COVID call, another COVID anxiety call. And she hung up on me, she said ‘that’s all I can do for you today, goodbye.’* Farhad

Some participants described strategies they had employed to secure referrals to specialists:

*‘I asked an ENT colleague, who said I should be referred to ENT, and I think that added a bit of weight to my request to be referred when I asked, because a specialist had recommended it.’* Irena

Those doctors who had been successful in being referred to specialist care also described experiencing delays in having their referrals actioned.

*‘I was sometimes asking for appointments every other day because we couldn’t get in touch with the hospital. The doctors there wouldn’t respond… It was literally a complete barrier.’* Harriet

Participants were upset and angry that they felt that they had been expected to work in the face of unknown risk of infection, not knowing there would be long‐term complications, and then not supported afterwards.

*‘And it was quite early on in COVID, so in our area, everybody, it was all a bit chaotic, the hot hub was only just established, everybody was just, you know, not that many cases of COVID, so I think it was quite hard for people and I think there was definitely a bit of, I don’t know, trying not, I don’t know like not wanting to engage with it because nobody really knew what to do.’* Jenna

*‘For a low risk patient we’ve returned to pre‐COVID levels of PPE… but that’s how I got ill, I got ill before we introduced PPE… I fear that I will become that neurotic doctor that refuses to not use PPE.’* Lisa

Those participants who had received support emphasized the value of being listened to and believed by a particular GP.

*‘I ask now to be phoned back by the person I’ve spoken to the most because I don’t have to repeat things and I know she believes me.’* Lisa

*‘Then I spoke to my normal GP when she got back and that was probably the single most helpful conversation that I had during all of this because she, I was really struggling with how bad the fatigue was… I couldn’t really have a shower without an hour’s sleep afterwards and was feeling absolutely awful. Just feeling really grotty all the time. And she completely validated that I wasn’t one of her nightmare patients.’* Jenna

### 3.3 Using medical knowledge and connections

Participants described how they were forced to self‐advocate because their own GPs were unsure how to investigate and manage their symptoms.

*‘*[My GP] *does rely heavily on me being a doctor and making my own management plan… There’s a place for ICE* [Ideas, Concerns and Expectations] *but I need someone to be my doctor. If I don’t come up with something, it’s “wait and see”, or a blood test.’* Irena

Participants described using their own connections to get referrals or advice from colleagues or friends.

*‘I’d messaged a friend from medical school who’s a cardiologist as I was wondering about pericarditis… I’ve always tried to be a good patient and go through my GP and things, but it wasn’t working. So that’s when I started messaging people and calling in favours.’* Georgina

Participants frequently went on to use those connections to gain input from the private sector, including investigations.

*‘My friend said “if you’ve got a mate in cardiology then ask for an echo”. So I did. And I don’t normally like to ask for favours… I reached out and he said “if you pay the fees for the echo then we’ll do it”… I felt disappointed I was unable to access this on the NHS. Something I pride myself in, I want to go the extra mile for my patients but I felt that I had hit resistance… You kind of give up trying, you think “why should I bother with this, I’ll just pay for it”, but then you fall into a vicious cycle where you’re paying for reassurance.’* Farhad

The disappointment in Farhad's reflection, about how the NHS had let him down, is evident and reflected in most participant narratives.

Some participants described trying complementary medicine to manage and improve their symptoms, despite recognizing the lack of evidence behind such therapies, and perhaps going against their previous evidence‐informed judgements.

*‘I have gone all out on the quackery, which I never thought I would because I've never been that person. I've had acupuncture a few times. I am under a dietician and I'm taking all sorts of weird and wonderful supplements.’* Deborah

### 3.4 Wanting to help and wanting to be helped

All participants, while wanting help for their own symptoms, also emphasized how they also wanted to raise awareness of long COVID. Participants reflected upon the therapeutic value of engaging in the interview process. Some doctors described other motivational factors including the desire to contribute their knowledge and insight based on lived experience although they were not currently working clinically.

‘*I had COVID and five months later, I’m still not better. In fact, things are still really quite difficult for me. I can’t look after my children for long periods of time, or in the initial stages at all, and that created huge problems. I’m just suffering a lot and I know that, from looking online, there are thousands of people who are similar out there. Up until very recently really, a lot of us haven’t had our voices heard.’* Alice

*‘I hope there will be a therapeutic value in telling my story.’* Georgina

All the participants reflected on the fragmented nature of their care so far. If they had been referred onwards, these were to different specialties, and participants described their own work in fitting everything together. Being doctors, they were able to reflect on what might be helpful for people struggling with persisting symptoms and suggested that a ‘one‐stop clinic’ was the optimal way of investigating people with long COVID. Most felt that this should be face‐to‐face including a physical examination. Such a service, however, would need to be tailored and personalized, not a ‘one‐size fits all’:

*‘What we need is to have a service where people can be referred to a central service whereby people listen to them and then do the screening… To screen the people who have symptoms suspicious of cardiac and respiratory issues that need further investigation.’* Edward

*‘I think it could be case‐by‐case… depending on the patient and what they’re presenting with. It might be for some people that a screening phone call would be enough but it might really vary.’* Lisa

However, some reflected that there could be a role for telephone or online support in addition to a face‐to‐face service:

*‘I think it’s a really good way of getting information out there, it just needs to be good quality information.’* Jenna

Jenna went on to reflect on the information available on the NHS ‘Your COVID Recovery’ website^38^:

*‘And it needs to be more accessible and more user friendly than the version that they’ve currently, the COVID service that is currently available online. And it’s also not very personalised, but that’s really difficult online.’* Jenna

Other participants who had looked at the ‘Your COVID Recovery’ NHS website^38^ said they felt it was not helpful or applicable to them, and worried that some of the advice given, particularly on graded activity, could cause them harm:

*‘They did pages on what poor concentration is but nothing about how to help. There is a lack of excluding significant pathology on that website. I’m not doing graded exercise because if that is giving me chest pain I don’t want to do it unless respiratory or cardiology say it’s OK. Pacing seems to be better. If I push myself like I did this week I pay for it. If it’s about getting your heart rate up I can do that by walking to the bathroom.’* Irena

### 3.5 Combining patient and professional identity

Participants frequently struggled with adjusting to their new dual identity as both doctor and patient.

*‘I have found it very difficult to dissociate my doctor brain from my patient brain. I found it very difficult to… I’m a trainer as well, and I found it very difficult to dissociate my educators’ brain from my patient brain so I’ve had that dynamic going on for several weeks. I said to him ‘I hope I’ve handed over that locus of control, I’m putting trust in you, you’re looking after me, I will go by your advice.’* Farhad

They also reflected on their new awareness of how the doctor‐patient relationship can be impacted by uncertainty around symptoms.

*‘As someone that's GP trained, I know what an absolute nightmare it is when a patient comes to you with 35 symptoms and it's completely overwhelming. I think I'm very aware of that and she always sounds like she's busy, stressed and wants to get off the phone.’* Alice

*‘It wasn’t an active prejudice, but in the back of my mind I hadn’t thought about it… a number of us on the group have said how ashamed we are of some of the attitudes we’ve had towards people, and lack of empathy… This concept of being irritated by patients when they’re not really pleased when something comes back normal… Hopefully it will make me a better and more empathetic doctor at the end.’* Kate

Participants felt that their experiences as a patient would help them be more supportive to people with difficult to explain symptoms when they returned to clinical practice.

*‘We were taught in medical school that in chronic disease the patient will know the disease the best… but I think this has really emphasized to me that listening to the patient story is the key to understanding what’s going on… and the symptoms might be the same or the textbook might say this is the main issue, but then the patient might experience it differently or it might not be the main issue for some patients… so listening more open‐mindedly and probably being a lot more humble… It’s so important as a patient to feel that even if they can’t fully understand what you’re going through that they do listen and try and understand and acknowledge.’* Lisa

## 4 DISCUSSION

### 4.1 Summary of findings

This is the first qualitative study reporting the perspectives of doctors experiencing persistent symptoms following infection with COVID‐19 in early 2020, as new knowledge was emerging. Doctors described the difficulty of making sense of unusual, unanticipated and persistent symptoms in the face of rapidly emerging and changing evidence (e.g. on NHS websites) and anecdote (e.g. on social media). Distress in the doctors’ narratives related to feelings of being ‘let down’ by their own doctors and the NHS, perceiving that they were not believed and having to consult frequently, emphasizing evidence of the severity of their illness, in order to be believed and to obtain specialist referral. Some doctors described using their own knowledge and personal connections to specialists to access care; others reported resorting to complementary therapies for the first time in the face of more traditional medical practices offering no solutions. Doctors had concerns about ongoing significant organ involvement and wanted such pathology to be excluded, having read about sequelae including myocarditis and pulmonary emboli following COVID‐19. These experiences emphasized a moral dilemma of wanting to be treated as a ‘patient’, yet, professionally, being dissatisfied with their care. All participants described the struggle of adjusting to their new dual identity as both doctor and patient and reflected on their worry that they would be seen as a ‘heartsink’ patient.^39^

Reasons for participating in this study included wanting to help others, to contribute to the growing body of evidence around long COVID, but also to voice their experiences, to be heard. Field notes taken contemporaneously all included reflections from participants that it had been ‘so helpful to have a chance to tell my story’. All the participants described how they also wanted help, to enable them and their doctors to understand their symptoms and be referred for investigations to look for underlying pathology. Most participants suggested that such help had not always been forthcoming. Participants reflected on how the experience of long COVID might lead them to be more empathic when meeting patients with difficult to understand symptoms in the future.

### 4.2 Strengths and limitations

While there is much discussion on social media and non‐peer‐reviewed publications,^6^, ^7^, ^8^, ^11^, ^12^, ^15^ this is the first qualitative study (embedded within a larger study^29^) that we are aware of that has focussed on the experience of doctors who have had persistent symptoms following COVID‐19 infection in early 2020.

Most participants were general practitioners, which reflects recruitment using social media and snowball sampling. Participants were predominantly white, female and in a younger age group which may reflect the likely preponderance of women with long COVID.^40^ The apparent homogeneity of the sample has implications for our claims of saturation and may be a limitation. While we feel our claims are justified for the themes identified, we acknowledge that alternative accounts from demographic groups not captured here could add novel insights and should be explored in further research.

Experts by experience (HA and TAB) were key members of the study team. In addition, other people with long COVID contributed to study design, ethics application, the patient information sheet and the interview topic guide. All study participants contributed to ongoing analysis, by providing comments on analysis summaries as analysis progressed.

### 4.3 Comparison with previous literature

There is a developing body of literature on long COVID, and the research team have published an earlier paper reporting analysis of 24 interviews.^29^ There are also patients’ narratives on social media and articles in journals, including from people with long COVID who are also doctors.^7^, ^12^

Physicians have previously described the ways in which their own illness has been ignored by the medical community, how their dignity and identity have been diminished through the experience of illness, poor communication which has left them upset and confused, and the perceived arrogance of the doctors managing them.^24^ Other reasons why doctors may be unwilling to seek help for mental health problems are stigma and the culture of invulnerability pervasive across medicine, which can result in asking for help being seen as ‘weak’ and ‘letting down the profession’.^23^ Doctors have in the past described themselves as ‘wounded healers’, borrowing Jung's term thought to derive from the ancient Greek legend of Asclepius who, in recognition of his own wounds, established a sanctuary at Epidaurus where others could be healed.^41^ Fox and colleagues interviewed 17 UK GPs who had experienced significant illness, and confirmed that the ethos of invulnerability to illness persists among GPs.^42^ Bradley reflects on the challenges that ill doctors face: their prior knowledge of the health system and personnel, possible concerns about the quality of care and difficulty finding the right GP.^43^ The narratives of the participants in our study reflect on all these barriers and describe how they have tried to overcome them.

Doctors in this study described how they utilized existing medical knowledge—regarding symptoms, treatments, care pathways and processes—and professional contacts with peers and services to navigate healthcare systems. These descriptions resonate with concepts of economic, social and cultural capital espoused by Bourdieu.^44^ Doctors in this research, arguably, sought to capitalize on their sense of social status by actualizing financial (seeking private healthcare, accessing alternative therapies), social (sense of social obligations within a network of professional peers) and cultural (embodied identity, objective status and accreditation as a doctor) resources to overcome barriers to health care commonly experienced by patients with medically unexplained symptoms. In doing so, doctors reproduced social inequalities experienced by those without access to such forms of capital and social position. However, given doctors’ descriptions of feeling let down, it seems apparent their sense of capital did not correspond, nor support, an improved sense of health care but instead informed reflective practice and motivation to adopt a more empathic approach in the future.

Previous studies suggest that when a doctor develops a condition, such as chronic fatigue syndrome/myalgic encephalomyelitis, they are more likely to believe in that condition subsequently.^45^ Doctors in this study reflected that they had a better understanding of the experiences of those who had ‘medically unexplained symptoms’ and they hoped to use their experience of COVID‐19 to benefit their clinical acumen when they returned to work, as well as making them more empathic to people with such symptoms.

Henderson et al's work into sick doctors returning to work included those with both physical and mental health problems, and reiterates the isolation and self‐stigma experienced by doctors with health problems.^27^ Medical training may create a doctor identity or ‘medical self’, which allows doctors to do their job effectively despite working long and stressful hours in the proximity of sickness and disease.^23^ Although these characteristics predominantly enable doctors to work productively, they can distort doctors’ ability to seek help when unwell and tolerate the role of a patient, and can make consultations challenging to negotiate due to unfamiliar power dynamics. When doctors are unwell, they may describe a conflicted sense of identity that, for some, manifested as a ‘loss of self’. It has previously been reported by doctors that their own identity has been taken over by ‘the role of the doctor’, and when they were unwell they attempted to reclaim their ‘sense of self’.^46^ The perception of doctors as ‘invulnerable’ can make it more challenging for them to return to work due to concern around negative responses and a lack of support from colleagues.^27^

The duration, full extent and severity of long COVID is not yet clear. Arnold reported that although patients with COVID‐19 may remain symptomatic at 8‐12 weeks, clinical abnormalities requiring action are infrequent, especially in those without a supplementary oxygen requirement during the acute phase of illness. It should be noted, however, that the COVERSCAN study suggested organ damage in 70% of patients that may need follow‐up or intervention; evolving evidence will clarify this.^18^ What is clear from the narratives of our participants is that doctors must play a significant role in validating the experience of those with long COVID, investigating worrying symptoms and offering emotional support while participants recover.

There is an argument that doctors with long COVID may need different or additional support because of the added complexities of navigating contractual issues and the challenge of returning to work (and its associated risk of exposure).^47^ The younger age (<45 years) of those who are typically affected by long COVID means that there are longer‐term workforce implications of the increase in doctors off sick. This not only may affect staffing levels in the ‘second wave’ of COVID‐19, but staffing levels in the coming months and potentially years following this as the timeline of recovery is as yet uncertain.^47^

### 4.4 Implications for research and practice

It is important to conduct further research to understand the characteristics of doctors who are more or less likely to be affected by long COVID, and cohort studies should explore the process and length of patient recovery. This will have implications for longer‐term workforce planning and developing ways to support affected doctors back into work. The experiences of doctors who treat other doctors with long COVID could also be explored.

As these doctors reflected, living with long COVID can be transformative and many expressed hope that some good will come of their experiences and challenges. This study may help to destigmatize illnesses where symptoms are as yet unexplained, such as long COVID, and study participants highlighted that the experience of illness would make them better able to care for and empathize with patients with ongoing and unexplained symptoms.

## CONFLICTS OF INTERESTS

The authors declare no competing interests.

## AUTHORS' CONTRIBUTIONS

CCG, HA and CO'D conceived the study. CCG wrote the protocol and obtained ethics approvals. AKT contributed to the protocol and production of analysis summaries for feedback to participants. CCG and TK conducted the interviews. CCG and AKT led the re‐analysis of data. AKT drafted the paper. All authors contributed to analysis and revising the paper. HA and TAB also contributed their expertise as people with long COVID.

## ETHICAL APPROVAL

The study received ethics approval from Keele University Ethics Committee (MH‐200134).

## PROVENANCE

Freely submitted; externally peer reviewed.

## Data Availability

Data available upon reasonable request to the authors.

## Footnotes

^1^ NHS 111 is a service, provided by the National Health Service (NHS), which allows people to access care either online or by telephone. The service is staffed by a range of staff who work through protocols in dealing with queries.

^2^ A triage site where patients with possible COVID‐19 can be referred for assessment—set up at the start of the COVID‐19 pandemic in England.
